# Supplementary material for: Significantly Improved COVID-19 Outcomes in Countries with Higher BCG Vaccination Coverage: A Multivariable Analysis
Source: Vaccines (Basel). 2020 Jul 11;8(3):378. doi: 10.3390/vaccines8030378 (PMC7563451; doi:10.3390/vaccines8030378)
Supplement: Supplementary file 1 [file vaccines-08-00378-s001.pdf]

Supplementary Online Content

Significantly Improved COVID-19 Outcomes in Countries with Higher BCG Vaccination Coverage: A Multivariable Analysis

Danielle Klinger[1], Ido Blass[2], Nadav Rappoport[3,*] and Michal Linial[1,*]

[1]Department of Biological Chemistry, Institute of Life Sciences, The Hebrew University of Jerusalem, Jerusalem, Israel [2]The Rachel and Selim Benin School of Computer Science and Engineering, The Hebrew University of Jerusalem, Jerusalem, Israel. [3]Department of Software and Information Systems Engineering, Faculty of Engineering Sciences, Ben Gurion University of the Negev, Beer Sheva, Israel

* Correspondence authors:

Supplemental Text S1. This includes supplementary methods and resources used in this study.

<u>**Figures S1-S2**</u>

- **Figure S1.** This includes the results of the correlation of BCG years of admission and COVID-19 outcomes for countries bounded by a population size of >100M.
- **Figure S2.** This includes the results from a multivariable analysis of country-centric data for countries bounded by a population size of >100M.

<u>**Tables S1-S5**</u>

- **Table S1.** Variable resources that are used across countries and populations
- **Table S2.** Additional resources on BCG administration years by country
- **Table S3.** Country cohort used in the study (55 countries)
- **Table S4.** Correlations and p-values of regression of years of BCG administrations and different outcomes including permutation tests
- **Table S5.** Multivariable results at numerous time points for BCG years of admission
- **Tables S6.** Sub-sampling of countries for years of BCG administration and COVID-19 outcomes

Text S1. Extended methods and procedures

COVID-19 information by country: Information regarding COVID-19 was extracted between January 29th, and May 21th, 2020 on a daily basis from the Worldometers website. The data fields included the following: Country, Total Cases, New Cases, Total Deaths, New Deaths, Total Recovered, Active Cases, Serious and Critical. Normalization (values per 1M) was calculated directly from the country-based report on population size. Data normalization was performed for each country by accounting for the relevant population size. We included in the analysis death per million (DPM), cases of hospitalization with serious and critical conditions (SPM). Other comparative measures include the recovered (RPM) and confirmed cases (CPM).

Demographic information by country: Demographic measures of countries were extracted from the Worldometers website on April 17th, 2020. The parameters extracted were the median age, life expectancy, percentage of urban population, and population estimate as compiled by the United Nations (UN). Information regarding the share of population above >65 years and economic development indicators were extracted from the World Bank data.

Comorbidities Information by country: Prevalence of smoking habits, health care capacity, handwashing facilities, extreme poverty and chronic diseases (e.g. obesity, type 2 diabetes, active tuberculosis and the death rate from cardiovascular disorders and cancer) were extracted from Our World In Data website which collect data from numerous reliable resources including the World Health Organization (WHO), the United Nations Statistics Division and the Institute for Health Metrics and Evaluation. Supplemental **Table S1** provides the source for each of the country-related information.

BCG vaccination information: Information regarding past and present BCG administration practices in every country was extracted from the BCG world atlas (see text). In order to establish the exact length and years of mandatory BCG administration, additional information was extracted from country-specific reports. The information is often shared by local health system authorities (often in local language). A detailed list of these resources is compiled in supplemental **Table S2**. In addition, the estimates for BCG vaccination coverage between years 1980-2018 were extracted from the annual WHO reports. In countries known to have a national vaccination policy, though without a known start date (8 of 55 countries), a *minimally assumed start year* was defined as the first year with >50% BCG coverage to newborns according to the WHO BCG vaccination reports. A *minimally assumed administration period* was determined for all countries, either with a known or assumed start date for the national vaccination BCG policy. The results reported in this paper only considers the countries with a strictly known BCG administration period. Nevertheless, the results prove to be true also for the group including the “minimally assumed administration period”. Both sets are available for analysis using our interactive website, listed as “strict”- for the countries with the strictly known administration period, or “imputed”- for the countries with the minimally assumed administration period.

General data analysis: Accounting for the varying time of the diseases across countries, we define a unified aligned key date of a country as the first date when DPM reached for the first time the value 0.5 or higher. The following analysis was conducted across changing dates (e.g. 10-50 days) for the key aligned date as defined. For country-specific groups (binary or categorized, such as BCG administration), we applied the ranked Wilcoxon test as a nonparametric statistical test for comparing any of the paired groups. For the continuous (i.e. the number of years BCG was administered, the prevalence of TB in the population, median age) data we applied linear regression, reporting the regression fit and the calculated statistical significance (p-value) for the COVID-19 outcomes.

Note on Norway's BCG administration years- The official mandatory BCG vaccination policy ended in 1997, however, the country established an elective vaccination policy that maintained high population turnouts (above 88% of babies were vaccinated) until 2010. For this reason, we considered Norway's administration period to be between 1947 and 2010.

Analysis according to age group coverage: The country population distribution by age was extracted from Our World in Data based on the UN, Department of Economic and Social Affairs, Population Division reports. The data was grouped into three age groups divided as 24 years and younger, 25 to 64, and 65 and older. The share of each age group out of the total population was calculated for each country. *BCG coverage year* was defined as the number of years in which a national mandatory BCG vaccination policy was in place. The number of years with BCG coverage was defined within each age group, out of the total number of possible years in that group. From the age partition and the BCG coverage, a value that measures the *percentage of the population in the share of the age group with BCG* is calculated. The final values in each country were processed through a linear regression algorithm opposing the variable of DPM. Countries without approved start year were excluded from the age group coverage analysis.

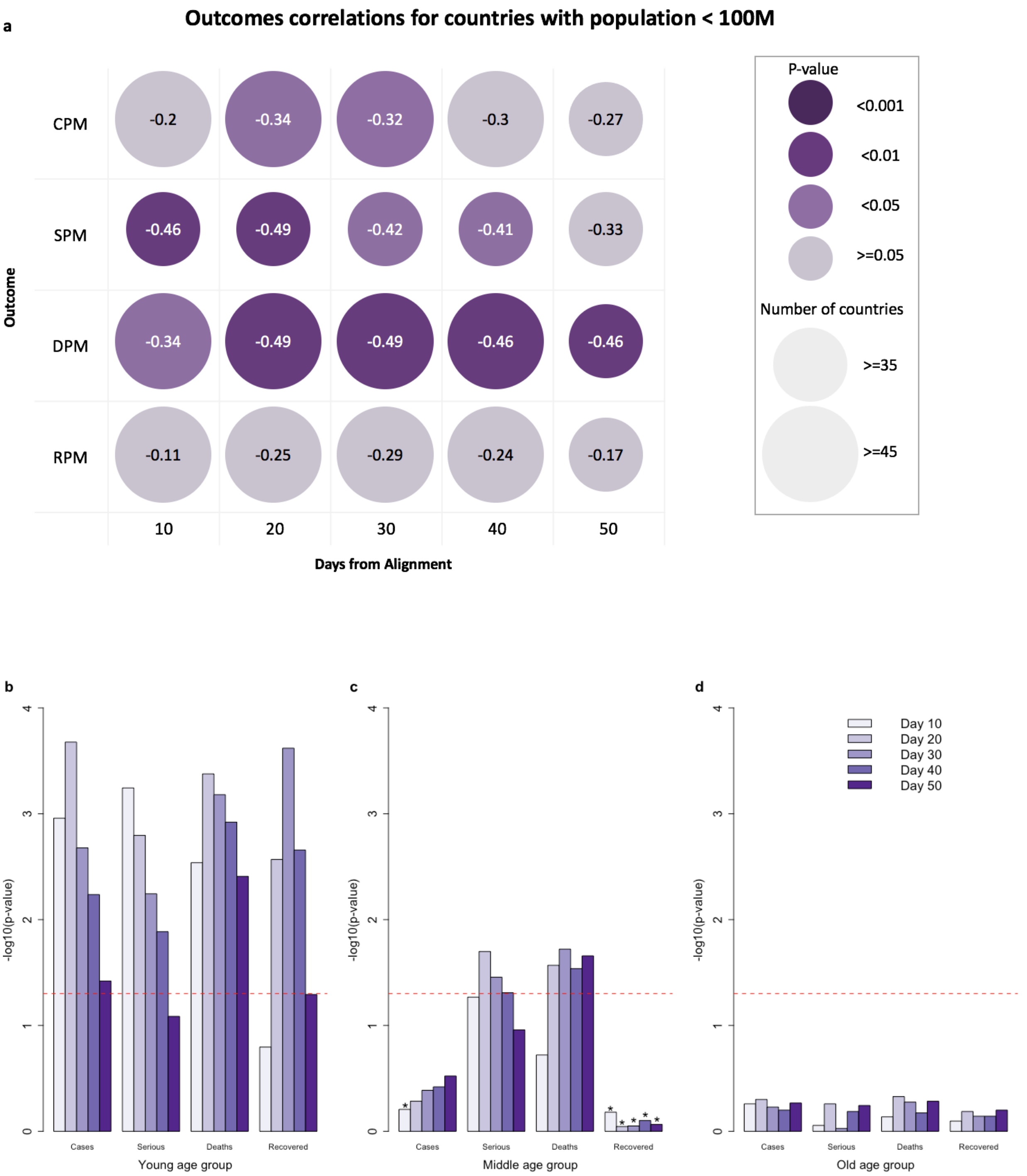
a
Outcomes correlations for countries with population < 100M
P-value
<0.001
<0.01
<0.05
>=0.05
Number of countries
>=35
>=45
Outcome
CPM
SPM
DPM
RPM
-0.2
-0.34
-0.32
-0.3
-0.27
-0.46
-0.49
-0.42
-0.41
-0.33
-0.34
-0.49
-0.49
-0.46
-0.46
-0.11
-0.25
-0.29
-0.24
-0.17
10
20
30
40
50
Days from Alignment
b
c
d
-log10(p-value)
Cases
Serious
Deaths
Recovered
Young age group
Middle age group
Old age group
Day 10
Day 20
Day 30
Day 40
Day 50

Figure S1. Outcomes correlations with years of BCG administered and coverage.

Each row represents a different COVID-19 outcome. Each column represents the time interval of the outcome from the alignment date. Countries included are bounded by population size <100M (total 50 countries). **(a)** Circle size depicts the number of countries, and color represents the statistical significance of the correlation with darker purple color indicating a higher significance. Values in circles are the correlation estimates where all the observed correlations are negative. Note that while all countries (55) reached 20 days post alignment of DPM >= 0.5, at 50 days post alignment, some countries (5) that were at an earlier phase of the pandemic failed to provide information. The histogram (b-d) shows the statistical significance of the correlation of BCG years of administration and the 4 different COVID-19 outcomes. Relative BCG coverage is partitioned to three age groups, weighted by population share: **(b)** young (0-24 years), **(c)** middle age (25-64) and **(d)** old age group (>65 years). Days from the key alignment date are colored from light to dark purple (10 to 50 days). The statistical significance is shown as -log10(p-value), the dashed red line indicates p-value of 0.05. Asterisk represents the outcome with a positive correlation. The outcomes with a positive correlation (marked by asterisks**)** are insignificant.

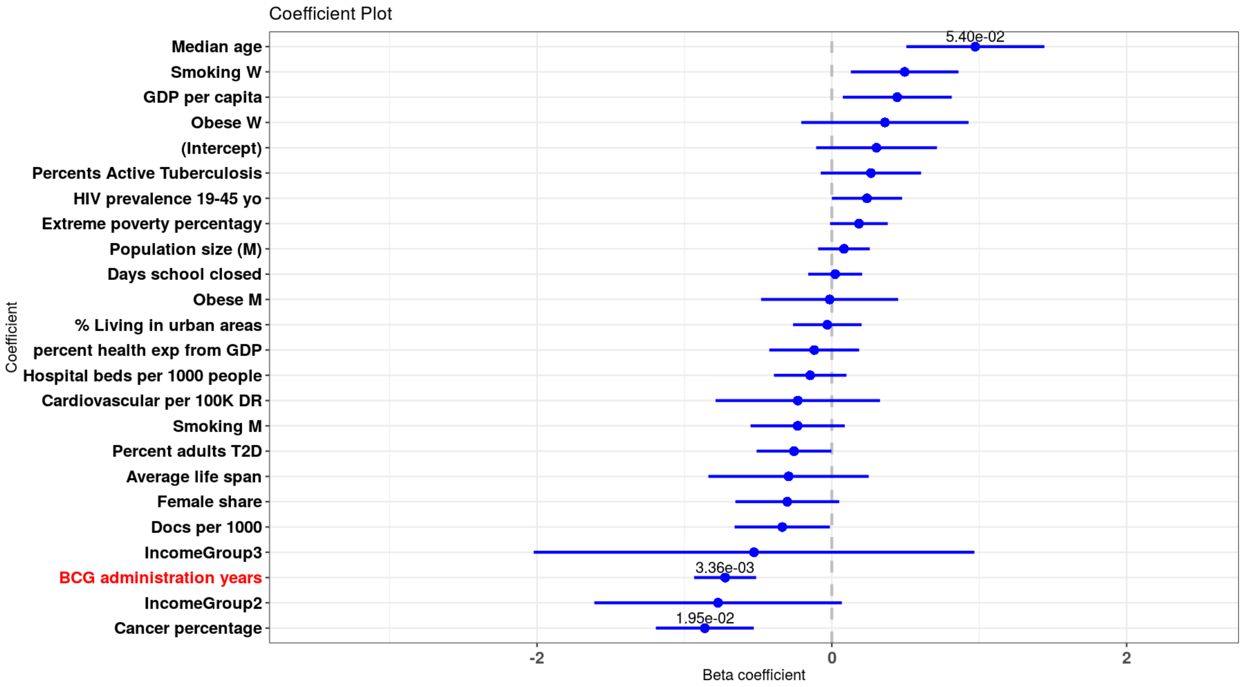

Figure S2. Multivariable analysis of country-centric quantitative data for 50 countries with population size <100M.

Beta coefficients of the normalized multivariable linear regression for DPM at day 20 are shown. Blue lines represent the coefficients' 95% confidence intervals. P-values were added for all variables with p-value < 0.1. The strongest significance is associated with BCG administration years (marked red).

Table S1. Variable resources used across countries and populations

Information extracted	Resource
**Physicians per 1000 people**	World Bank, (2016), *Physicians include generalist and specialist medical practitioners,* Published online at OurWorldInData.org. Retrieved from: 'https://ourworldindata.org/grapher/physicians-per-1000-people?tab=chart' [Online Resource].
**Hospital beds per 1000 people**	OECD; Eurostat; World Bank; National Government Records and other sources. (1960-2018). *Hospital beds per 1,000 people, 1960 to 2018.* Published online at OurWorldInData.org. Retrieved from: 'https://ourworldindata.org/grapher/hospital-beds-per-1000-people' [Online Resource].
**Share of men who smoke**	World Bank. (2016). *Share of men who smoke, 2016.* Published online at OurWorldInData.org. Retrieved from: 'https://ourworldindata.org/grapher/share-of-men-who-are-smoking' [Online Resource].
**Share of women who smoke**	World Bank. (2016). *Share of women who smoke, 2016.* Published online at OurWorldInData.org. Retrieved from: 'https://ourworldindata.org/grapher/share-of-women-who-are-smoking' [Online Resource].
**Diabetes prevalence**	World Bank. (2017). *Diabetes prevalence, 2017.* Published online at OurWorldInData.org. Retrieved from: 'https://ourworldindata.org/grapher/diabetes-prevalence' [Online Resource].
**Proportion of population with basic hand washing facilities**	United Nations Statistics Division. (2017). *Proportion of population with basic handwashing facilities on premises, 2017.* Published online at OurWorldInData.org. Retrieved from: 'https://ourworldindata.org/grapher/proportion-of-population-with-basic-handwashing-facilities-on-premises' [Online Resource].
**Share of population living in extreme poverty**	World Bank. (2017). *Share of the population living in extreme poverty, 1981 to 2016.* Published online at OurWorldInData.org. Retrieved from: 'https://ourworldindata.org/grapher/share-of-the-population-living-in-extreme-poverty' [Online Resource].
**Share of women that are obese**	World Health Organization. (2016). *Share of women that are obese, 2016.* Published online at OurWorldInData.org. Retrieved from: 'https://ourworldindata.org/grapher/share-of-females-defined-as-obese' [Online Resource].

**Share of men that are obese**	World Health Organization. (2016). *Share of men that are obese, 2016.* Published online at OurWorldInData.org. Retrieve from: 'https://ourworldindata.org/grapher/share-of-men-defined-as-obese' [Online Resource].
**Share of people living with active Tuberculosis**	Institute for Health Metrics and Evaluation. (2018). *Share of people living with active tuberculosis, 2017.*Published online at OurWorldInData.org. Retrieved from: 'https://ourworldindata.org/grapher/share-of-people-living-with-active' [Online Resource].
**Share of population infected with HIV within the 15-49 age group**	Institute for Health Metrics and Evaluation . (2018). *Share of the population infected with HIV, 2017.*Published online at OurWorldInData.org. Retrieved from: 'https://ourworldindata.org/grapher/share-of-population-infected-with-hiv-ihme' [Online Resource].
**Death rate from cardiovascular disease**	Institute for Health Metrics and Evaluation. (2018). *Death rate from cardiovascular disease, 2017.*Published online at OurWorldInData.org. Retrieved from: 'https://ourworldindata.org/grapher/cardiovascular-disease-death-rates' [Online Resource].
**Share of population with cancer**	Institute for Health Metrics and Evaluation. (2018). *Share of population with cancer, 2017.* Published online at OurWorldInData.org. Retrieved from: 'https://ourworldindata.org/grapher/share-of-population-with-cancer' [Online Resource].

Table S2. Additional resources on BCG administration years by country

Country	Additional resources on BCG administration years
Algeria	Moline M, B. P. (1950), *First results of a year of BCG vaccinations in Algeria,* Alger Medicale.
China	Chen ZR, W. X. (1982), *BCG in China,* Chinese Medical Journal (English).
Greece	Gabriele, F., Katragkou, A., & Roilides, E. (2014), *BCG vaccination policy in Greece: time for another review?* International Union Against Tuberculosis and Lung Disease.
Malaysia	Arnold Loh, L. J. (2019), *BCG unable to prevent TB in adults.* Report published online at thestar.com. Retrieved from 'https://www.thestar.com.my/news/nation/2019/09/19/bcg-unable-to-prevent-tb-in-adults' [Online Resource].
Morocco	Bouazzaoui, Naima Lamdouar (2006*), Évolution du calendrier vaccinal au Maroc,* Bulletin de L'Académie Nationale de Médecine.
Norway	Report from Norway's Medicines Agency. Published online at fhi.no. Retrieved from: 'https://www.fhi.no/nettpub/vaksinasjonsveilederen-for-helsepersonell/vaksiner-mot-de-enkelte-sykdommene/tuberkulosevaksinasjon---veileder-f/' [Online Resource].
Panama	Pinzon, T. P. (1953), *BCG Vaccination in the Republic of Panama,* American Review of Tuberculosis.
Philippines	Published report, (1988), Expanded Programme on Immunization – Philippines, Virus Inf Exch Newsl South East Asia West Pac.
Portugal	Report published in the Vaccine European New Integrated Collaboration Effort. Published online at http://venice.cineca.org. Retrieved from: 'https://www.venice.cineca.org/documents/portugal_ip.pdf' [Online Resource].
Serbia	D, Pesut. (2004), *Contemporary status of BCG vaccine in the world and in Serbia,* Medicinski pregled.
Tunisia	Cyrine Bennasrallah, M. K. (2019), *BCG vaccination and tuberculosis prevention: A forty years cohort study, Monastir, Tunisia,* PLOS One.

Table S3. Country cohort used in the study

Country	Region	Population size (M)		Country	Region	Population size (M)
**Algeria**	Africa	44		**Italy**	Europe	60
**Argentina**	Latin America	45.2		**Lebanon**	Middle East	7
**Australia**	Oceania	25.5		**Malaysia**	South-East Asia	32.3
**Austria**	Europe	9		**Mexico**	Latin America	129
**Belarus**	Europe	9.4		**Moldova**	Europe	4.0
**Belgium**	Europe	11.5		**Morocco**	Africa	36.9
**Bolivia**	Latin America	11.6		**Netherlands**	Europe	17.1
**Bosnia and Herzegovina**	Europe	3.3		**Norway**	Europe	5.4
**Brazil**	Latin America	213		**Panama**	Latin America	4.3
**Bulgaria**	Europe	7		**Peru**	Latin America	33
**Canada**	North America	37.7		**Philippines**	South-East Asia	110
**Chile**	Latin America	19		**Poland**	Europe	37.9
**China**	East Asia	1439.3		**Portugal**	Europe	10.2
**Colombia**	Latin America	50.8		**Romania**	Europe	19.2
**Croatia**	Europe	4		**Serbia**	Europe	8.7
**Czechia**	Europe	10.7		**Slovakia**	Europe	5.5
**Denmark**	Europe	5.8		**South Korea**	East Asia	51.2
**Dominican Republic**	Latin America	10.8		**Spain**	Europe	46.7
**Ecuador**	Latin America	17.5		**Sweden**	Europe	10.0
**Finland**	Europe	5.5		**Switzerland**	Europe	8.6
**France**	Europe	65.2		**Tunisia**	Africa	11.8
**Germany**	Europe	83.8		**Turkey**	Middle East	84.3
**Greece**	Europe	10.4		**Ukraine**	Europe	43.73
**Honduras**	Latin America	9.9		**United Arab Emirates**	Middle East	10
**Hungary**	Europe	9.6		**United Kingdom**	Europe	67.8
**Iran**	Middle East	84		**United States**	North America	331
**Ireland**	Europe	4.9		**Uruguay**	Latin America	3.4
**Israel**	Middle East	8.6				

Table S4: Correlations and p-values of regression of years of BCG administrations and different outcomes

Outcome	Days post alignment	# of countries included	Correlation	p-value	Permutation p-value*
CPM	10	47	-0.23	0.1132	0.1265
CPM	20	47	-0.38	**0.0091**	**0.0085**
CPM	30	47	-0.36	**0.0133**	**0.0120**
CPM	40	46	-0.34	**0.0196**	**0.0105**
CPM	50	42	-0.31	**0.0488**	0.051
SPM	10	40	-0.43	**0.0052**	**0.0065**
SPM	20	40	-0.48	**0.0018**	**0.0020**
SPM	30	40	-0.42	**0.0065**	**0.0035**
SPM	40	39	-0.44	**0.0069**	**0.0070**
SPM	50	36	-0.35	**0.0354**	**0.0370**
DPM	10	47	-0.34	**0.0195**	**0.0215**
DPM	20	47	-0.48	**0.0006**	**0.0000**
DPM	30	47	-0.49	**0.0005**	**0.0010**
DPM	40	46	-0.46	**0.0013**	**0.0001**
DPM	50	42	-0.45	**0.0025**	**0.0025**
RPM	10	46	-0.09	0.5732	0.5725
RPM	20	46	-0.23	0.1308	0.1385
RPM	30	45	-0.27	0.0777	0.0870
RPM	40	43	-0.22	0.1610	0.1690
RPM	50	39	-0.14	0.3963	0.3910

* P-values are given as correlation p-values in view of 2000 permutations' p-values for Pearson’s correlation.

Table S5. Ranking of the 'BCG administration years' variable within the multivariable regression analysis

Days post alignment	p-value	P-value rank	Beta coefficient	Number of countries
10	0.00186	1	-0.65	47
15	0.00084	1	-0.52	47
20	0.00194	1	-0.74	47
25	0.00335	1	-0.75	47
30	0.00346	1	-0.71	47
35	0.00478	1	-0.71	47
40	0.00994	1	-0.67	46
45	0.0142	1	-0.64	46
50	0.0583	4	-0.52	42

Table S6: Sub-sampling p-value at different COVID-19 outcomes.

Outcome	Days post alignment	# of countries included	Correlation	p-value	% of significant Sub-samples[a]
CPM	10	47	-0.23	0.1132	2.4
CPM	20	47	-0.38	**0.0091**	**95.4**
CPM	30	47	-0.36	**0.0133**	85.4
CPM	40	46	-0.34	**0.0196**	75
CPM	50	42	-0.31	**0.0488**	34.8
SPM	10	40	-0.43	**0.0052**	**95.4**
SPM	20	40	-0.48	**0.0018**	**99.3**
SPM	30	40	-0.42	**0.0065**	**97.1**
SPM	40	39	-0.44	**0.0069**	**98.6**
SPM	50	36	-0.35	**0.0354**	53.5
DPM	10	47	-0.34	**0.0195**	75.5
DPM	20	47	-0.48	**0.0006**	**99.9**
DPM	30	47	-0.49	**0.0005**	**99.6**
DPM	40	46	-0.46	**0.0013**	**99**
DPM	50	42	-0.45	**0.0025**	**98**
RPM	10	46	-0.09	0.5732	0
RPM	20	46	-0.23	0.1308	2.9
RPM	30	45	-0.27	0.0777	11.6
RPM	40	43	-0.22	0.1610	4.9
RPM	50	39	-0.14	0.3963	0.9

[a]Out of 2000 sub-sampling tests, sampling 90% of the countries. In bold face all cases in which the results are statistically significant.
